# Nanopatterning of Weak Links in Superconducting Oxide Interfaces

**DOI:** 10.3390/nano11020398

**Published:** 2021-02-04

**Authors:** Gyanendra Singh, Edouard Lesne, Dag Winkler, Tord Claeson, Thilo Bauch, Floriana Lombardi, Andrea D. Caviglia, Alexei Kalaboukhov

**Affiliations:** 1 Department of Microtechnology and Nanoscience—MC2, Chalmers University of Technology, SE 412 96 Gothenburg, Sweden; singhgy@chalmers.se (G.S.); dag.winkler@chalmers.se (D.W.); tord.claeson@chalmers.se (T.C.); thilo.bauch@chalmers.se (T.B.); floriana.lombardi@chalmers.se (F.L.); 2 Kavli Institute of Nanoscience, Delft University of Technology, Lorentzweg 1, 2628 CJ Delft, The Netherlands; E.F.Lesne@tudelft.nl (E.L.); a.caviglia@tudelft.nl (A.D.C.)

**Keywords:** LaAlO_3_/SrTiO_3_ interface, two-dimensional superconductivity, top-down lithography, nanopatterned materials

## Abstract

The interface between two wide band-gap insulators, LaAlO${}_{3}$ and SrTiO${}_{3}$ (LAO/STO), hosts a quasi-two-dimensional electron gas (q2DEG), two-dimensional superconductivity, ferromagnetism, and giant Rashba spin-orbit coupling. The co-existence of two-dimensional superconductivity with gate-tunable spin-orbit coupling and multiband occupation is of particular interest for the realization of unconventional superconducting pairing. To investigate the symmetry of the superconducting order parameter, phase sensitive measurements of the Josephson effect are required. We describe an approach for the fabrication of artificial superconducting weak links at the LAO/STO interface using direct high-resolution electron beam lithography and low-energy argon ion beam irradiation. The method does not require lift-off steps or sacrificial layers. Therefore, resolution is only limited by the electron beam lithography and pattern transfer. We have realized superconducting weak links with a barrier thickness of 30–100 nm. The barrier transparency of the weak links can be controlled by the irradiation dose and further tuned by a gate voltage. Our results open up new possibilities for the realization of quantum devices in oxide interfaces.

## 1. Introduction

Complex oxide interfaces have recently been recognized as a powerful platform to engineer and study novel electronic phases [1]. Advances in thin film growth techniques have provided routes to artificial (hetero)structures and emergent phases that are inaccessible by traditional chemical synthesis, and have allowed the properties of existing materials to be modified on the atomic scale [2,3,4,5]. The discovery of a two-dimensional electron gas (2DEG) that exhibits superconductivity, co-existing with intrinsic ferromagnetism and large gate-tunable spin-orbit coupling, in the interface between two wide band-gap insulators, LaAlO${}_{3}$ and SrTiO${}_{3}$ (LAO and STO), has stimulated increasing interest in both experimental and theoretical studies of this system [6,7,8,9,10,11,12,13,14]. In particular, the combination of two-dimensional superconductivity with multiband occupation and strong Rashba spin-orbit coupling in the LAO/STO interface is very promising in a realization of superconducting pairing with unconventional order parameter [15,16,17,18].

Reliable fabrication of nanostructures with dimensions comparable with the superconducting coherence length $\xi_{0}\approx$ 50 nm is necessary for quantum devices based on the LAO/STO interface and studying fundamental superconducting properties. Direct patterning of the LAO/STO is remarkably challenging as the materials are very inert to standard etching processes [19]. Ion beam etching is routinely used for nanostructure patterning in complex oxides, but remains particularly difficult to control. Indeed, in case of the LAO/STO interface, ion beam etching may create oxygen vacancies in the STO substrate, and these donate electrons to the interface and modify its electrical transport properties, potentially creating undesired electrical shorts between or within devices. [20]. Therefore, alternative routes to circumvent these problems have been developed. A direct writing using a conducting-tip atomic-force microscope (CAFM) allows fabrication of nanostructures with lateral dimensions as small as 2 nm [21]. That writing mechanism is based on surface protonation in the presence of H${}_{2}$O which dissociates into OH${}^{-}$ and H${}^{+}$ under a biased tip [22]. However, the ionized surface is crucially depending on thickness, limited to the insulator-metal transition occurring between 3 and 4 unit cell thick LAO, and suffers from time instability due to relaxation processes after writing. In another approach, an amorphous sacrificial layer of the LAO has been used to pattern interfaces without using ion beam etching [23,24]. A protective 2 unit cell thick epitaxial LAO film is first deposited on the STO substrate. This does not result in a formation of an electron gas at the interface. After this, an amorphous LAO film is deposited at room temperature using a lift-off mask. After lift-off of the amorphous LAO layer, a second epitaxial LAO film is deposited. This creates a metallic interface in the selected areas, where the amorphous film was removed. This method allowed fabrication of superconducting nanowires with dimensions down to 200 nm [25]. In our previous work, we developed another patterning method based on low-energy Ar${}^{+}$ ion beam irradiation of the LAO/STO interface [26]. Using a low-energy Ar${}^{+}$ ion beam is crucial to avoid formation of oxygen vacancies in the STO substrate. At the same time, short irradiation of several minutes eliminates electrical conductivity at the interface without physical removal of the LAO film. The metal-insulator transition induced by irradiation has been attributed to the Ar ion implantation in the LAO film [27,28]. Such direct patterning is advantageous as it does not require any sacrificial lift-off layers and the resolution is only limited by the electron beam lithography patterning of the mask used during the irradiation. Using this method, we have successfully fabricated high-quality superconducting nanowires with dimensions down to 50 nm [29,30].

While lateral electronic confinement can be successfully realized by several methods, fabrication of weak links in the LAO/STO interface is much more challenging. Weak links are key elements for investigation of the superconducting pairing mechanism through the measurements of current-phase relationship [31,32]. So far, there are only a few reports on the practical realization of weak links in the LAO/STO interface. A combination of global back-gate and local top-gate has been used to create short overdamped weak links [33] that displayed critical current oscillations as a function of magnetic field, resembling a Fraunhofer pattern due to the dc Josephson effect. However, due to stray fields, the top gate approach is limited in realizing short weak links comparable with the coherence length of the LAO/STO ξ≈50 nm. In another approach, a split-gate configuration was used to induce superconducting-to-insulating transition and create a gate voltage controlled Josephson effect [24,34]. Alternatively, lithographically defined Dayem bridges, with channel width and length of about 200 nm, have been realized using an amorphous template technique [25]. A grain boundary approach to create weak links in high-temperature superconductors has been widely used. However, it is not applicable to the LAO/STO interface as its conductivity strongly depends on scattering at dislocations and rapidly decreases for grain boundary angles above 10 degrees [35].

Here, we present an alternative approach to fabricate artificial weak links in the LAO/STO superconducting interface by using a modified low-energy Ar${}^{+}$ ion irradiation technique [26,27,29]. We utilize the possibility to control the interface conductivity by adjusting the dose of the Ar${}^{+}$ ion irradiation. In a double-step fabrication, a uniform channel is first patterned using high dose Ar${}^{+}$ ion irradiation, while the weak links are created in the second step by lower dose Ar${}^{+}$ ion irradiation through a narrow slit across the channel. In this way, one can independently tune the size and transparency of the weak link. This technique allows realization of weak links with a minimum barrier thickness of about 30 nm while preserving elsewhere the superconducting transport properties of the pristine LAO/STO regions.

## 2. Materials and Methods

LaAlO${}_{3}$ films were grown on TiO${}_{2}$-terminated STO substrates by ablating a single crystal LAO target using pulsed laser deposition (PLD) (λ = 248 nm) with laser fluence of 1.0 J/cm${}^{2}$ and repetition rate 1 Hz. The substrate was heated to a temperature of 500 ${}^{\circ }$C and film was deposited in an oxygen partial pressure p${}_{O2}$ = 10 ${}^{-4}$ mbar. The epitaxial growth was monitored using in situ reflection high-energy electron diffraction (RHEED). The RHEED showed clear intensity oscillations confirming layer-by-layer growth. After the growth, film was annealed at 600 ${}^{\circ }$C in an oxygen pressure ∼300 mbar to compensate for oxygen vacancies created during growth and which could lead to extrinsic conduction.

Electrical contacts (5 nm Ti and 100 nm Au) were fabricated by lift-off technique and dc magnetron sputtering. This method provides low-ohmic contact resistance to the LAO/STO interface [26,27]. Electron beam lithography was performed using a high-resolution electron beam lithography system, JEOL JBX-9300FS (JEOL Ltd., Tokyo, Japan). Low-energy Ar ion beam irradiation was performed in an Oxford Instruments IonFab 300 Plus system (Oxford Instruments Plasma Technology, Bristol, UK) using Kauffman Ar${}^{+}$ ion source with 7 cm beam aperture. The resistance of the sample was monitored in real-time during irradiation process using a Keithley K2400 (Keithley Instruments Inc., Cleveland, OH, USA) source meter in two-probe configuration. The ion beam was switched off at each measurement point to avoid electrical interference. The process was terminated when resistance reached ∼20 MΩ to prevent overexposure of the interface.

I–V characteristics and other transport measurements were performed in a dilution refrigerator (Oxford Triton, Oxford Instruments Nanoscience, Oxford, UK) equipped with a combination of mu-metal shields and a superconducting lead shield to give protection from background magnetic field noise, resulting in a residual field of less than 100 nT. The external noise was suppressed by using twisted pairs of superconducting NbTi/Cu lines in combination with two stages of cryogenic filters. A Cu powder filter was installed at the base temperature stage of the refrigerator, together with low-pass RC filters with a cutoff frequency of 0.2 MHz at the 4 K stage. The noise was further filtered by using conventional electromagnetic interference filters at room temperature. Room temperature low noise filtered amplifiers were used for conventional four-probe measurements of the I–V characteristics.

## 3. Results

### 3.1. Nanopatterning of Weak Links

**Figure 1:**
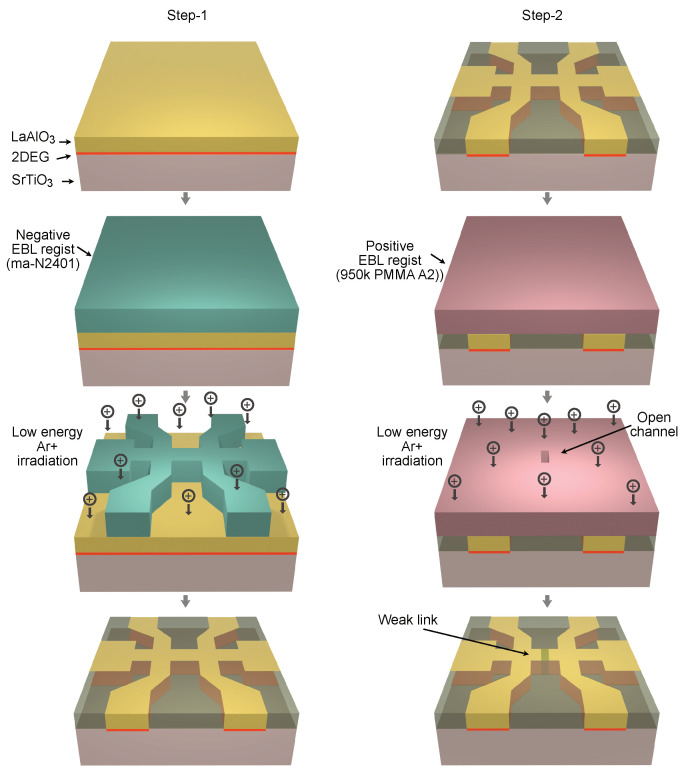
Two-step fabrication process for weak links in the LAO/STO interface. In the first step, narrow channels are created by electron beam lithography and low-energy ion irradiation. In the second step, the weak links are created by low dose Ar${}^{+}$ ion irradiation through a slit across the prefabricated channel using electron beam lithography and a positive e-beam resist.

**Figure 2:**
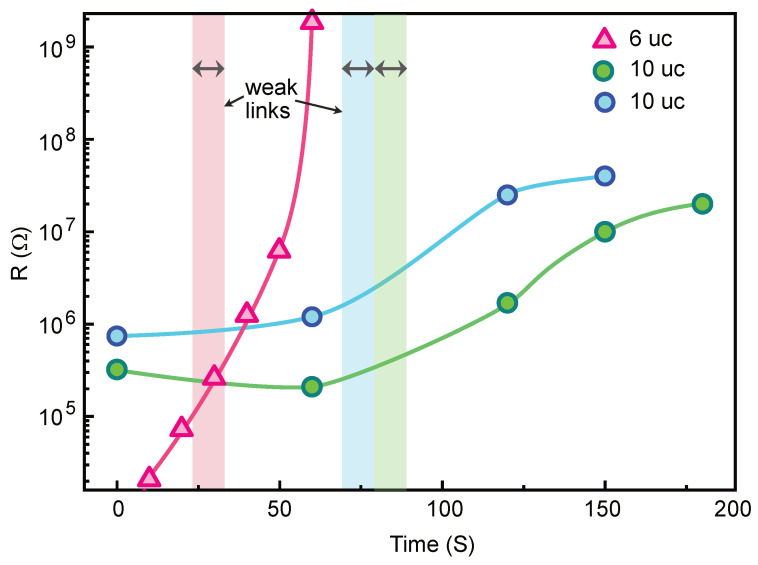
Electrical resistance as a function of Ar${}^{+}$ ion beam irradiation time for three different LAO/STO samples (one 6 uc and two 10 uc thick) patterned using electron beam lithography and the negative e-beam resist ma-N2401. All samples were cleaned in RF oxygen plasma for 10 s prior to the irradiation process. Vertical bars indicate the time interval used in the second Ar${}^{+}$ ion irradiation of weak links. The electrical resistance was measured in situ during the ion irradiation process in a two-probe configuration. The Ar${}^{+}$ ion beam acceleration voltage was 150 V, and the ion current was 30 μA/cm${}^{2}$.

**Figure 3:**
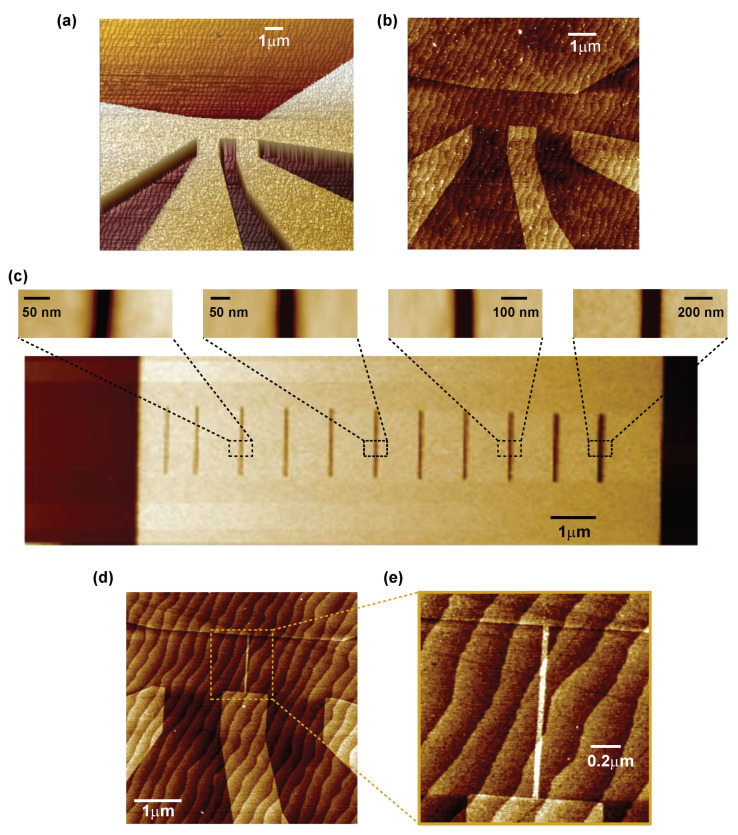
(**a**) AFM topography 3D image of the negative resist template after first electron beam lithography. Resist thickness is 60 nm. (**b**) AFM topography image of the same structure after ion beam irradiation and resist removal. (**c**) AFM topography image of open slits with a width ranging between 20 and 140 nm which were fabricated in positive e-beam resist after dose optimization in the second electron beam lithography. (**d**) AFM image of the final device containing a weak link with barrier thickness 30 nm. (**e**) Enlarged region of the AFM image shown in (**d**) containing weak link structure. Darker regions correspond to conducting paths, whereas light regions are highly resistive and insulating areas.

Uniform channels and weak links were patterned in 5 and 10 uc thick LaAlO${}_{3}$ (LAO) thin films deposited on TiO${}_{2}$ terminated (001)-SrTiO${}_{3}$ (STO) substrates. Details of film growth are presented in the Methods section. The carrier concentration and electron mobility of our LAO/STO pristine films were found to be of order $2\times 10^{13}$ cm${}^{-2}$ and 3000 cm${}^{2}/$Vs, respectively, in the pristine state (prior to gating) at 1.5 K. The fabrication process consists of two steps; see Figure 1. In the first step, narrow channels with line width of 100 nm to 1 μm are fabricated by electron beam lithography and a negative tone resist (ma-N2401 from Micro Resist Technology GmbH, Berlin, Germany) with a thickness of 60 nm. Among several negative tone resists, ma-N2401 has the advantage of relatively high resolution capability (≈50 nm) and is easy to remove from the surface after the development process. The latter is an important feature for device patterning in the LAO/STO interface as presence of surface contamination may significantly alter the carrier concentration and electron mobility of the electron gas [36,37]. In addition, the irradiation process is also very sensitive to surface contamination. The presence of a residual resist layer results in the increased irradiation time. After e-beam exposure and development of the resist, the sample is irradiated by an Ar${}^{+}$ ion beam. As we have shown earlier, using a low energy beam voltage below 150 eV is crucial to avoid formation of oxygen vacancies in the LAO/STO interface that induce additional charge carriers [26,27]. Two minutes of irradiation is sufficient to render the interfaced formed by 10 uc LAO on STO completely insulating. We always monitor the resistance of the sample in situ during the irradiation to avoid overexposure. Figure 2 shows the dependence of interface resistance on an irradiation time. One can fine tune the interface resistance between the metallic and insulating states despite the very short irradiation time. We also found that the actual irradiation time that is required to achieve an insulating state varies from sample to sample. This has been attributed to surface contamination of the sample after resist development. Therefore, obtaining curves similar to those presented in the Figure 2 is important for the reliable fabrication of different samples with desired weak-link characteristics. Using oxygen plasma cleaning for 5–10 s also improves the reproducibility of the irradiation process. However, oxygen plasma cleaning has to be used with care as it is known to increase the interface resistance [38]. This is a reason why the initial resistance of samples in Figure 2 is in the range of ∼200–600 kΩ/□, while typical sheet resistance of the LAO/STO interface is about ∼50 kΩ/□. Another disadvantage of the oxygen plasma cleaning is that it also etches the resist itself at a rate of ≈5 Å/s, which changes the dimensions of the structures. The insulating state induced by Ar${}^{+}$ ion irradiation is stable at room temperature for several years, but the metallic state can be recovered by annealing at high temperature above 550 ${}^{\circ }$C [27]. Figure 3a shows a 3D topography AFM image of a 1.3 μm channel negative resist mask after development. The same channel after Ar${}^{+}$ ion irradiation is shown in Figure 3b. Remarkably, the irradiated areas have higher profile in the topography AFM image. This is a result of a swelling effect of the LAO film due to the Ar ion implantation and partial surface amorphization that increases the volume of the material [27,28]. The surface of the film is also very clean with atomic terraces clearly visible in both irradiated and protected areas. We emphasize that no additional cleaning procedure is required after resist removal.

After fabrication of the narrow channels, the sample was cleaned and covered again by the positive tone electron beam resist MCC NANO 950k PMMA A2 (Kayaku Advanced Materials, Inc., Westborough, MA, USA) with thickness of 50 nm. This resist has very high resolution capability but suffers from low dry etching resistance. Nevertheless, it is sufficient to withstand the short Ar${}^{+}$ ion irradiation time needed in our process. In the second electron beam lithography step, a small opening was created in the positive resist; see Figure 1. The designed width of the opening slit in the resist was in the range of 15–100 nm. After this, a second Ar${}^{+}$ ion irradiation was applied with smaller dose as indicated by vertical lines in the Figure 2.

Fabrication of weak links with dimensions below 50 nm requires careful calibration of the electron beam dose on each sample. We attribute this to different properties of the STO substrates that result in different electron scattering, whereas proximity effect is negligible due to very small dimensions of exposed areas. Figure 3c shows the AFM image of calibration structures in the positive resist after dose optimization. Using this procedure, we can reliably fabricate lines with width of 15 nm in the resist. After ion irradiation, however, the line width is increased due to slow etching of the resist during ion beam irradiation. Figure 3d shows the final fabricated device with channel line width of 1.3 μm and nominal weak link barrier thickness of 15 nm. The weak link is clearly visible in the AFM image (Figure 3e) due to the swelling effect with actual thickness of the barrier estimated to about 30 nm.

### 3.2. Electrical Transport Properties in Normal and Superconducting States

**Figure 4:**
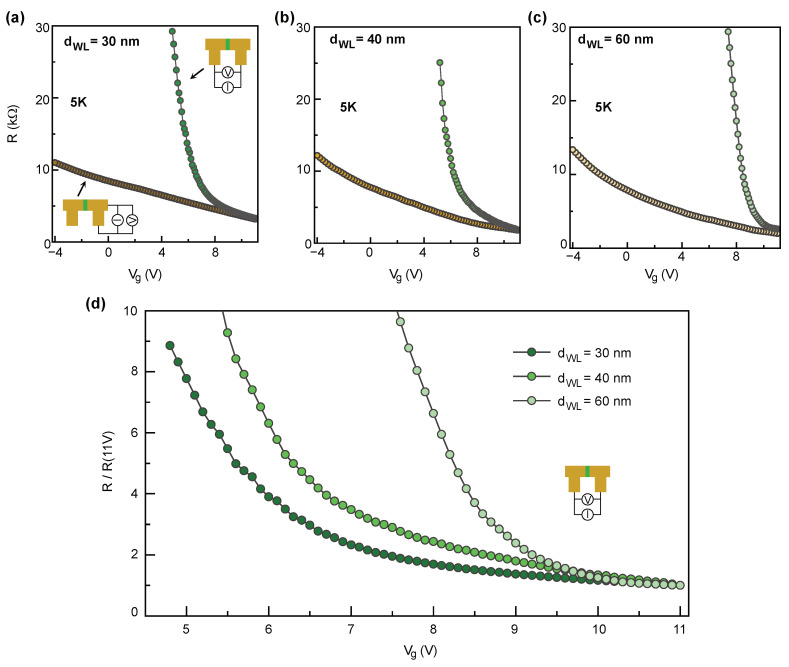
Normal state properties. (**a**–**c**) Normal resistance of three devices with barrier thickness d${}_{WL}$∼30, 40, and 60 nm, as a function of gate voltage for weak links (green data point) and uniform channels (yellow data point) measured in two point configuration measured at T = 5 K. (**d**) Comparison of the evolution of resistance as a function of gate voltage measured across the weak link. The resistance is normalized to the values corresponding to the maximum gate voltage in all panels, R(V${}_{max}$).

First, we investigated transport properties of fabricated devices in the normal state. Figure 4a–c shows dependence on the global back gate voltage of the resistances across the weak links with different barrier thickness and of a uniform part of the nanowire measured at 5 K in two point configuration. The resistance of the weak link increases much faster as compared to the uniform channels when the gate voltage is decreased from +11 to +5 V. This indicates that the weak links have indeed much higher resistivity as compared with the electrodes. Figure 4d shows comparison of normalized resistance across the weak links with different barrier thickness as a function of back gate voltage. The resistivity of the weak links increases with barrier thickness that further proves the feasibility of the fabrication approach for weak links.

**Figure 5:**
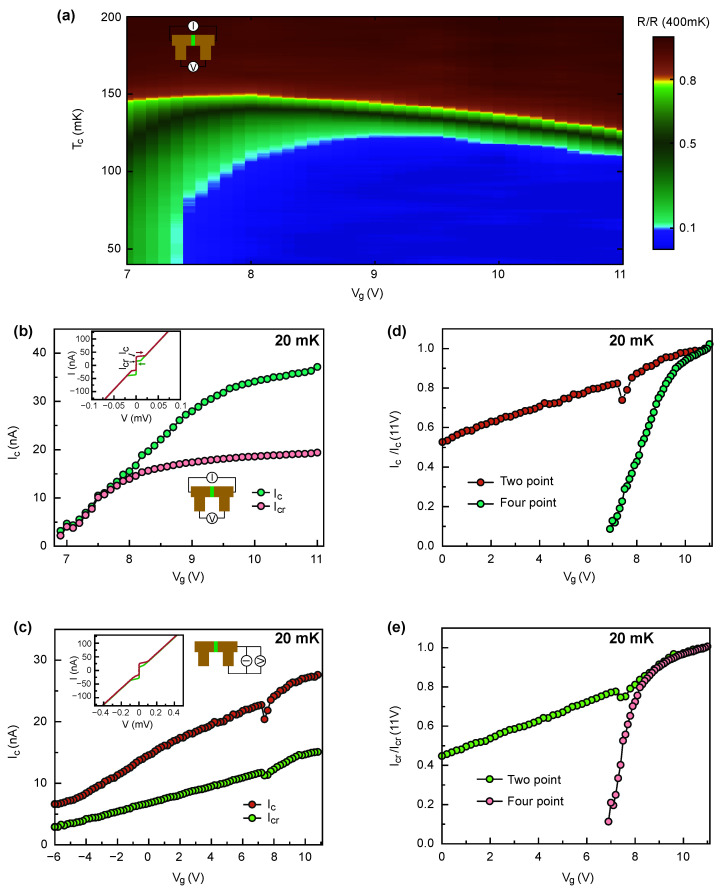
Superconducting properties of weak link devices. (**a**) Color plot of a resistance of the weak link device with barrier width 30 nm as a function of temperature and gate voltage. The resistance is normalized to the resistance at 400 mK. (**b**) The evolution of average critical current ($I_{c}=\frac{I_{c}^{+}-I_{c}^{-}}{2}$) and average return current ($I_{cr}=\frac{I_{cr}^{+}-I_{cr}^{-}}{2}$) as a function of gate voltage for the weak link device measured in four-probe configuration. The I${}_{c}$ and I${}_{cr}$ are identified as switching current, in I–V curves, to normal state in forward bias, and returning current to superconducting state respectively. Inset shows the I–V curve of the device measured at 11 V. (**c**) The gate voltage dependent I${}_{c}$ and I${}_{cr}$ for the uniform device measured in two point configuration. Inset shows the I–V curve of the device at 11 V. The comparison of normalized average critical current (**d**) and normalized average returning current (**e**) as a function of gate voltage.

Figure 5a shows a color plot of the resistance normalized to the resistance at 400 mK for the weak link device with barrier thickness of 30 nm as a function of temperature and gate voltage. The maximum critical temperature is about 150 mK. The temperature dependence of the resistance does not show the presence of an additional transition that can be associated with the weak link barrier, indicating that the weak link barrier remains non-superconducting within the whole range of gate voltages. Figure 5b,c shows dependencies of critical and return currents extracted from measured I–V characteristics (shown in insets of Figure 5b,c) as a function of gate voltage for the weak link and the uniform part of the channel, correspondingly. Weak links were measured in four-probe configuration, while two probe configuration was used for uniform channels. Measurements were performed at the base temperature of the dilution refrigerator of 20 mK. The critical current of the weak link is suppressed much faster the one of the uniform channel. This agrees with gate voltage ranges obtained from normal resistance measurements; see Figure 4. Figure 5d,e shows the gate voltage dependence of the normalized critical current and return current for each configuration. Interestingly, the return current of the weak link device follows the one of the uniform channel and is suppressed only when the critical current becomes very low.

## 4. Discussion and Conclusions

We have developed a patterning method for the controlled fabrication of artificial weak links in superconducting oxide interfaces of LAO/STO that does not involve sacrificial layers or lift-off process as used in the amorphous template technique. Direct high-resolution electron beam lithography allows fabrication of weak links with minimum barrier thickness of about 30 nm, which is comparable (or smaller) than the superconducting coherence length of the LAO/STO interface. The resolution is mainly limited by the low resistance of the positive e-beam resist to the ion beam irradiation. Using harder e-beam resists should improve the resolution but may also affect the electrical transport properties of the interface due to surface contamination.

We have also demonstrated that weak link devices’ electrical properties are clearly distinct from uniform bridges by the gate voltage dependence of the critical current and normal resistance. This implies that the superconducting transport in our weak-link devices is dominated by the irradiation dose and width of the weak-link, and can not be solely attributed to a speculative inhomogeneous superconducting ground state. An important question is about the nature of our weak links. From the results of irradiation experiments, the weak links are not expected to be completely insulating but their resistance is higher as compared to that of the metallic electrodes at room temperature. We intentionally irradiated weak links during the second fabrication step for a relatively short time, as illustrated in Figure 2. This results in an approximately ten fold increase in the normal resistance. Since the weak link part is very short, its total contribution to the electrical resistance in the normal state is negligible. The fact that all devices show finite conductivity in the normal state even at low temperatures indicates that the barrier remains conductive upon cooling without strong freezout effect. Since it is unlikely that irradiated areas are superconducting, the weak links remain in the normal state below the superconducting transition of the electrodes. Upon decreasing the gate voltage from +11 V, the resistance of the weak link rapidly increases and superconducting coherence disappears resulting in the absence of critical current. Therefore, the behavior of our weak link devices is likely described by superconductor-normal metal-superconductor (SNS) junctions. More detailed analysis of the behavior of our devices is complicated by the inhomogeneous nature of the electron gas at the LAO/STO interface, both in the normal [39,40,41,42] and the superconducting states [43,44,45,46]. Both theory and experiments suggest the superconducting condensate in the LAO/STO consists of superconducting islands weakly connected through disordered metallic regions [44,47]. Therefore, the superconducting properties of our devices are likely a combination of intrinsic inhomogeneities and artificial weak links created by nanopatterning.

Further understanding of the nature of our weak links requires measurements of critical current as a function of magnetic field (to prove the dc Josephson effect) and under microwave irradiation (to prove the ac Josephson effect). We have already performed measurements of the critical current in perpendicular magnetic field that show anomalous enhancement of the critical current at low magnetic fields. This behavior suggests presence of Josephson channels with intrinsic phase shifts, possibly due to an unconventional pairing in the LAO/STO superconductor [48]. The very high dielectric constant and high loss of the STO substrate make measurements of Shapiro steps cumbersome.

## Data Availability

The data in this study are available from the corresponding author on request.

## Abbreviations

The following abbreviations are used in this manuscript:

RHEED	Reflection High Energy Electron Diffraction
PLD	Pulsed Laser Deposition
EBL	Electron Beam Lithography
AFM	Atomic Force Microscopy
